# Protocol for the evaluation of a quality-based pay for performance scheme in Liberia

**DOI:** 10.1186/s13012-014-0194-9

**Published:** 2015-01-13

**Authors:** Luke Bawo, Kenneth L Leonard, Rianna Mohammed

**Affiliations:** Ministry of Health and Social Welfare, Monrovia, Liberia; Department of Agricultural and Resource Economics, University of Maryland, College Park, MD 20740 USA; The World Bank, Washington, DC USA

**Keywords:** Liberia, Impact evaluation, Performance-based financing, Results-based financing, Pay for performance, Health-care quality

## Abstract

**Background:**

Improving the quality of care at hospitals is a key next step in rebuilding Liberia’s health system. In order to improve the efficiency, effectiveness, and quality of care at the secondary hospital level, the country is developing a system to upgrade health worker skills and competencies, and shifting towards improved provider accountability for results, including a Graduate Medical Residency Program (GMRP) and provider accountability for improvements in quality through performance-based financing (PBF) at the hospital level.

**Methods/design:**

This document outlines the protocol for the impact evaluation of the hospital improvement program. The evaluation will provide an estimate of the impact of the project and investigate the mechanism for success in a way that can provide general lessons about the quality of health care in low-income countries. The evaluation aims 1) to provide the best possible estimate of program impact and 2) to quantitatively describe the changes that took place within facilities as a result of the program. In particular, the impact evaluation focuses on the changes in human resources within the hospitals. As such, we use a three-period intensive evaluation of treated and matched comparison hospitals to see how services change in treated hospitals as well as a continuous data collection effort to track the activities of individual health workers within treated hospitals.

**Discussion:**

We are particularly interested in understanding how facilities met quality targets. Did they bring in new health workers with higher qualifications? Did they improve the knowledge or competence of their existing staff? Did they improve the availability of medicines and equipment so that the capacities of existing health workers were improved? Did they address the motivation of health workers so that individuals with the same competence and capacity were able to provide higher quality? And, if they did improve quality, did patients notice?

## Background

The civil war destroyed Liberia’s health system. Much of the physical infrastructure and equipment that were crucial to the health sector were destroyed during the war—many hospitals and clinics were burned to the ground; very few county hospitals had fully functional laboratories; most county hospitals and health centers were without running water, electricity, or functioning basic sanitary systems; and many health professionals, especially physicians, left the country. The latter resulted in a severe shortage of human resources. An already dire situation was further aggravated by a lack of transportation and other communication systems.

Despite impressive gains in overall health systems management and in health services delivery since the end of the war [1], Liberia continues to face significant challenges in improving maternal and child health outcomes, as well as other health-related Millennium Development Goal (MDG) outcomes [2]. Post-conflict conditions place Liberia at the bottom of global rankings for maternal and child health. The maternal mortality ratio remains high, but has declined from close to 1,000 per 100,000 births in 2007 to an estimated 770 per 100,000 in 2010. Gains, however, remain skewed in favor of urban populations. For example, 63% of deliveries in urban areas are facility-based compared with 25% in rural areas; similarly, 77% of urban deliveries are by a skilled service provider compared with only 32% of rural deliveries. While over one in ten children will die before the age of five, infant and under-five mortality rates have almost halved to 71 and 110 per 1,000 births, respectively, over the last 20 years due to improved access resulting from the Government of Liberia’s (GOL) free health-care policy and restoration of a number of key child health services like immunizations. Malaria, however, continues to be a major source of morbidity and mortality; 38% of outpatient attendance and 42% of inpatient deaths were attributable to malaria in 2007.

Improving the quality of care at hospitals is a key next step in rebuilding Liberia’s health system. Hospitals in Liberia remain in generally poor physical condition; are staffed with insufficient numbers of productive, responsive, and qualified staff in key areas of competence [3]; have long waiting times; and lack equipment and drugs [4]. As a consequence, hospitals in general provide low quality of care. This is reflected in high levels of post-surgery complications and infection rates, low-quality data on clinical outcomes, very limited maternal and child death audits, and no systematic use of clinical guidelines and protocols. Poor quality is a particularly critical concern at the severely resource-constrained hospital level in Liberia because it can obviate the implied benefits of good access and effective treatment, frustrate the positive achievements at the primary health-care system by not being able to respond to referral patients with complications, and lead to sub-optimal and wasteful use of resources [5].

In order to improve the efficiency, effectiveness, and quality of care at the secondary hospital level, the country is developing a system to upgrade health worker skills and competencies and shifting towards improved provider accountability for results. The Ministry of Health and Social Welfare (MoHSW) is tasked to develop a Graduate Medical Residency Program (GMRP) to facilitate in-country specialization of core MDG-related hospital-level competencies. Residents will be selected from the existing pool of medical school graduates based on standardized criteria. This process requires both a critical stream of specialist faculty to support the program, as well as the upgrading of teaching facilities. In addition to the development of an MDG-related GMRP, the GOL is also moving towards provider accountability for improvements in quality through performance-based financing (PBF) at the hospital level. The shift towards PBF is influenced by experiences in a number of high-, middle-, and low-income countries that performance-based approaches, in which providers receive incentives based on performance, can improve provider accountability for improved quality of health services.

### The project development objective

The project development objective of the Liberia Health Systems Strengthening Project (HSSP) is to “improve the quality of maternal health, child health, and infectious disease services in selected secondary-level health facilities”. The project aims to strengthen the institutional capacity needed to improve maternal health, child health, and infectious disease-related health outcomes at target facilities through an innovative approach involving systematic and coordinated improvements to the quality of services delivered at target facilities (through performance-based incentives) and an expansion of health worker skills (through the provision of specialized training and decentralization of specialist availability). Specifically, the project will (a) focus on improving the quality of care standards (in both diagnosis and treatment) for services with proven effectiveness; (b) increase the availability of qualified graduate physicians (pediatricians, obstetricians, general surgeons, and internal medicine specialists, with cross-cutting focus on anesthesiology); (c) enhance the clinical capabilities and competencies of mid-level cadres—nurses, midwives, and physician assistants—in emergency obstetrics, surgery, pediatrics, and internal medicine; and (d) improve provider-accountability mechanisms related to both the achievement of results and health worker performance at selected facilities. These improvements should provide a thrust towards improved outcomes.

The HSSP is focused on improving the quality of care in a number of services, outlined in Table 1. Note that the program only incentivizes quantities for a few numbers of services because, as hospitals, the goal is not to attract patients away from more appropriate locations but to provide the best care for those patients who need hospital care. However, although the PBF aspect of the program focuses on these services, the levers of the HSSP will affect a much broader array of services. In particular, the program increases the skills of health workers through training, improves the equipment and infrastructure levels of the hospitals directly as well as through increased availability of funds through PBF, and increases the motivation of health workers directly through the possibility of bonuses in PBF as well as improved management and information. These changes could lead to improvements in the quality of services and, in addition, to increases in utilization of key services.

**Table 1:** **Services addressed by PBF**

**PBF services**	**Definition**
Quality of complicated and assisted pregnancy and delivery (including C-section)	Any labor that is made more difficult or complex by a deviation from the normal procedure. Complicated delivery is defined as assisted vaginal deliveries (vacuum extraction or forceps), C-section, episiotomy, and other procedures.
Quantity of normal deliveries for at-risk referrals	High-risk pregnant women referred by health center to the hospital but delivered normally. A high-risk pregnancy is defined as evidence of edema, malpresentation, increased BP, multi-parity, etc.
Quantity of counter referral letters returned to health centers	Hospital returns counter referral letters with feedback on the referred patient to the referring health center. The counter referral letter is completed in triplicate, with one also given to the patient and one retained by the hospital.
Quantity of newborns referred for emergency neonatal care treatment	Newborns referred for emergency neonatal care due to perinatal complications, low birth weight, congenital malformation, asphyxia, etc.
Quantity of referred under-fives with fever	Infants and under-fives with fever who were referred to the hospital for management of malaria and pneumonia.
Quality of minor surgical intervention	Any surgical procedure that does not involve anesthesia or respiratory assistance.
Quality of major surgery (excluding CS, including major trauma)	Any surgery in which the patient must be put under general spinal/anesthesia and given respiratory assistance. Major surgery in the case of this package of services is defined as any of the following: herniorrhaphy, appendectomy, myomectomy, splenectomy, salpingectomy, hysterectomy, thyroidectomy, and mastectomy.
Quantity of patients transported by ambulance	Patients transferred from a lower-level facility (health center or health clinic) to the hospital for emergency treatment.

### Theory of change

**Figure 1:**
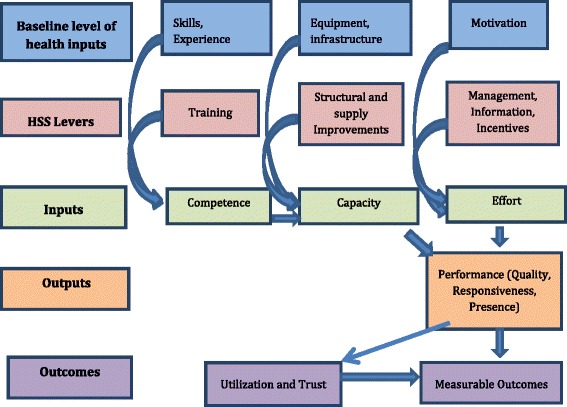
**Theoretical framework: theory of change.**

Figure 1 diagrammatically demonstrates the theory of change linking changes in skills, equipment, and motivation brought about by the levers of the HSSP and utilization. The skills and experience of health workers determine their *competence*; competence can be increased by training. The equipment and infrastructure available to health workers, combined with their competence determines their *capacity*; capacity can be improved by changes in competence or by structural and supply improvements to the facility. Finally, health worker *effort*, combined with capacity, determines the *performance* of health workers. Effort is driven by motivation and can be improved through better management, increased information, and/or improved incentives. Performance improves measurable outcomes directly and indirectly through increased utilization of health care services from patients who are more likely to trust the services provided.

### Impact evaluation design and methods

The HSSP will take place in five previously selected hospitals. Given the small number of hospitals in Liberia, it was determined at early stages that a full randomized controlled trial was not feasible. Thus, for the evaluation at the hospital level, the impact evaluation takes the project as a given, without control over the selection of treated or control hospitals and the timing or structure of any interventions. Five control facilities have been chosen to match the treated hospitals. The original criteria were that hospitals be drawn from urban and rural areas, from different regions of Liberia and from public and private services. Thus, each control facility is matched to the paired treatment facility on these characteristics. Facilities were not selected on the basis of initial quality levels or capacity to absorb the program.

These restrictions on the number of hospitals suggest a simple before and after matched pair comparison using measurable outcomes directly and indirectly tied to the project. However, with only five units in treatment and control groups, this strategy has very limited power to detect statistically significant improvements in outcomes. Although we will report these outcomes, the evaluation strategy focuses, instead, on the theory of change within treated facilities and a series of augmented PBF interventions at the health worker level within treated facilities. The external validity of the program as a whole is limited because the design is specific to Liberia, but the lessons about how things change within hospitals should have more generalizable findings.

The evaluation ((574034–2) Health Systems Strengthening Project (Liberia) Evaluation) was reviewed by the Institutional Review Board of the University of Maryland, College Park, and was approved on 5/16/2014. We will seek the consent of the management team at control hospitals while treatment facilities are required to participate in the evaluation as part of the project. All health workers in either kind of facility have the right to refuse to be observed, interviewed or evaluated and will sign forms if they choose to consent. All patients and caregivers can refuse to be observed or interviewed and the evaluation team must obtain oral consent from all patients or caregivers before collecting data on the services provided. No information that could be used to identify patients will be collected.

### Evaluating the theory of change within HSSP facilities

In order to understand better how PBF (as defined by the HSSP) is functioning within hospitals, we will collect the data on competence, capacity, and performance. Since these three measures are necessarily linked (performance is a function of capacity and capacity is a function of performance), we will focus on the differences between these three measures or the “three gaps” for health workers in treated and control facilities.

#### The three-gap framework

**Figure 2:**
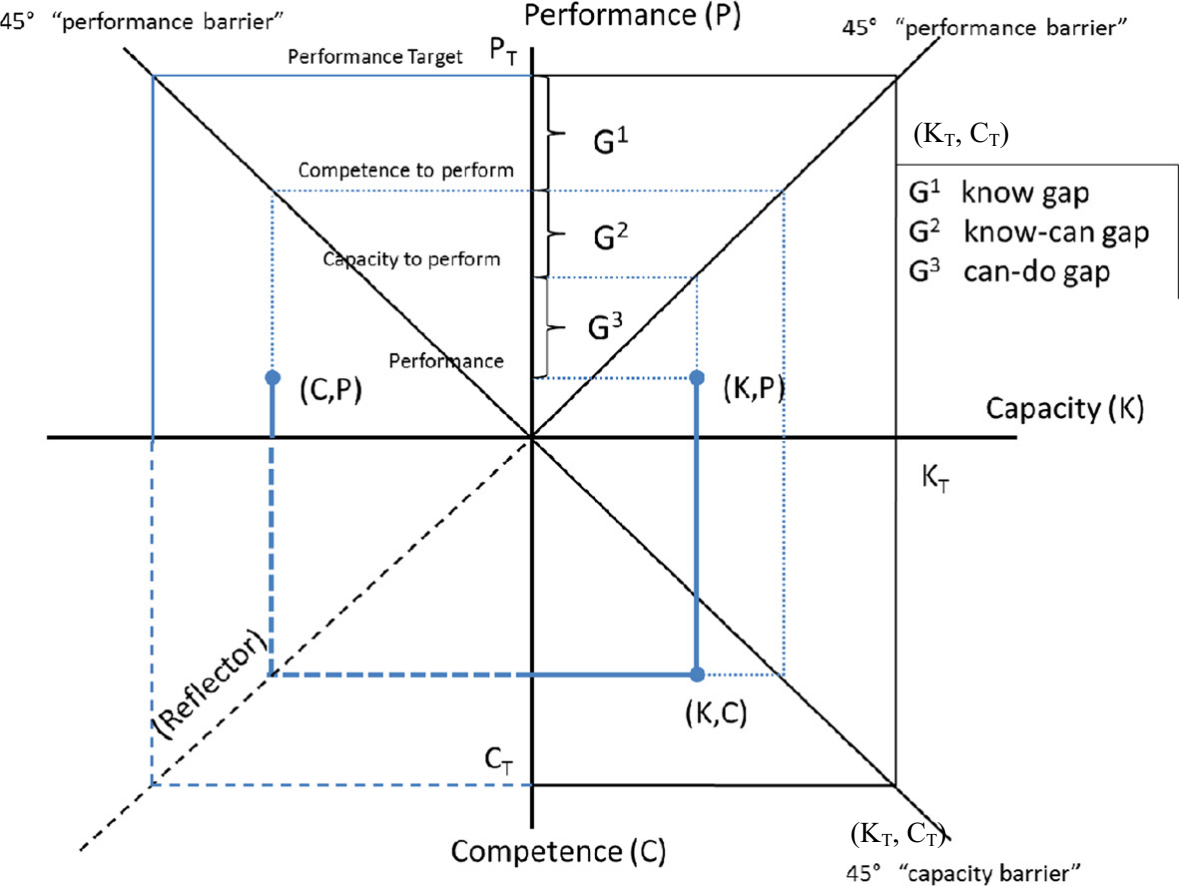
**The three-gap framework.**

The three-gap framework focuses on four levels of care: 1) the competence to perform, 2) the capacity to perform, 3) actual performance, and 4) the target levels of performance. The three gaps defined by these four levels are the know gap, the know-can gap, and the can-do gap. Figure 2 is a graphical demonstration of the three-gap framework. Performance originates with knowledge, education, and skills as measured by competence (C), shown on the vertical axis extending below the origin: increases in competence are shown by points closer to the bottom of the figure (further from the origin). Capacity (K) comes from competence taking into account the infrastructure, equipment, and medicines necessary to appropriately use training, education, and skills, shown on the horizontal axis to the right of the origin. Finally, performance comes from taking capacity and combining it with effort: health workers must choose to use their knowledge and equipment in order to perform, shown on the vertical axis above the origin.

An important feature of this model is the fact that performance is limited by capacity and capacity is limited by competence: a health worker cannot do better than what he or she knows how to do, for example. This is shown in the graph by what we call the capacity and performance barriers. In the translation from competence to capacity and again from capacity to performance, we take into account the capacity barrier and performance barrier, shown as 45° lines from the origin in the lower right and upper right quadrants. These barriers reflect the fact that better equipment cannot produce capacity beyond the level of competence and greater effort cannot produce performance beyond the level of capacity. In other words, competence limits capacity and capacity limits performance.

Target competence is the level required to perform according to the target level of performance, marked in Figure 2 as C_T_. In the ideal world, any health worker with target competence would also have target capacity K_T_: if health workers had all the equipment and medicines to work according to their training, capacity and competence would be the same. In addition, this capacity (K_T_) would ideally translate directly into targeted performance, P_T_.

The idea of the three-gap framework is that, in the real world, not all health workers have target competence, competence does not always translate into capacity, and capacity does not always translate into performance. In addition to this ideal triplet (C_T_, K_T_, P_T_), Figure 2 shows an example of another possible triplet (C, K, P). Competence (C) is lower than targeted performance and because some equipment or medicine is not available, this particular health worker has capacity (K) which is lower than his competence. This is shown by the pair (K, C) in the lower right quadrant and the fact that (K, C) is to the left of the capacity barrier. Furthermore, because effort is not ideal, the health worker does not fully transform capacity into performance (P), as shown by the pair (K, P) in the upper right quadrant. Thus, performance is significantly below target performance. Importantly, we can divide the short fall into three gaps:

1. the know gap (shown as G^1^), which is the difference between targeted performance and the competence to perform,
2. the know-do gap (shown as G^2^), which is the difference between competence and capacity and
3. the can-do gap (shown as G^3^), which is the difference between capacity and performance.

We also examine a slightly simplified two-gap framework. It is possible to reduce the three gaps down to two gaps and this is also shown in Figure 2. By using the lower left quadrant as a “reflector”, we can measure competence on the horizontal axis going to the left from the axis. The competence performance pair is shown in the upper right quadrant as (C, P). In such a case, we can measure the “know-do” gap which is also the sum of the know-can and can-do gaps.

Note that these gaps are important for policy reasons. A large know gap suggests deficiencies in training; a large know-do gap suggests deficiencies in infrastructure, equipment, or medicines; and a large can-do gap suggests deficiencies in motivation. However, the size of the gaps by themselves is not enough to understand the potential gains from changes in policy. In order to do this, we need to understand the relationship between competence, capacity, and performance.

**Figure 3:**
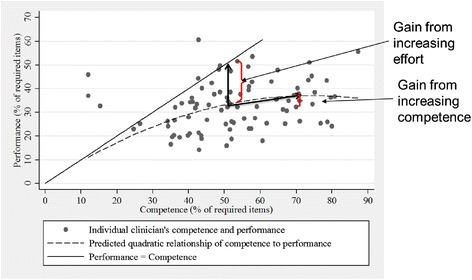
**Empirical evidence of the know-do gap.** Sources: [6,7]. The data show the measured competence and performance of a sample of clinicians. If clinicians followed their training, every data point would be near the 45° line, where competence is equal to performance. The dashed line shows the estimated relationship for the whole sample.

Focusing only on the know-do gap, we can look at some examples from available evidence and the important policy implications of this evidence. Figure 3 presents data on a sample of clinicians from urban and rural Tanzania whose competence and performance were measured. This graph is the mirror image of the upper left quadrant in Figure 2. Note that four health workers have performance above their competence, which is not supposed to happen—real-world data are always somewhat messy. In addition, a significant number of clinicians are grouped near the performance barrier, as should be expected. However, most clinicians are well below the performance barrier.

Performance in this setting is low: clinicians only do about 35% of what they are supposed to do for their patients. One apparent reason for this low performance is that competence or knowledge is low: clinicians only know about 50% of what they are supposed to know. So, it seems that competence needs to be improved. However, the data reveal a more complex relationship between competence and performance. The dashed line on the graph shows the relationship between competence and performance over the whole sample. For the clinician with average competence, increasing competence by 20 percentage points (from 50% to 70%) increases performance by only three percentage points (from 33% to 36%), a very small gain. On the other hand, any policy that takes competence as a given and closes the gap between competence and performance (the know-do gap) would see an improvement of up to 17 percentage points.

Thus, for the average clinician in this sample, improving competence does not improve performance altering the way we understand potential policies. Findings like these have led to a shift from a focus on competence to a focus on the know-do gap (see for example, [6,8-12]).

**Figure 4:**
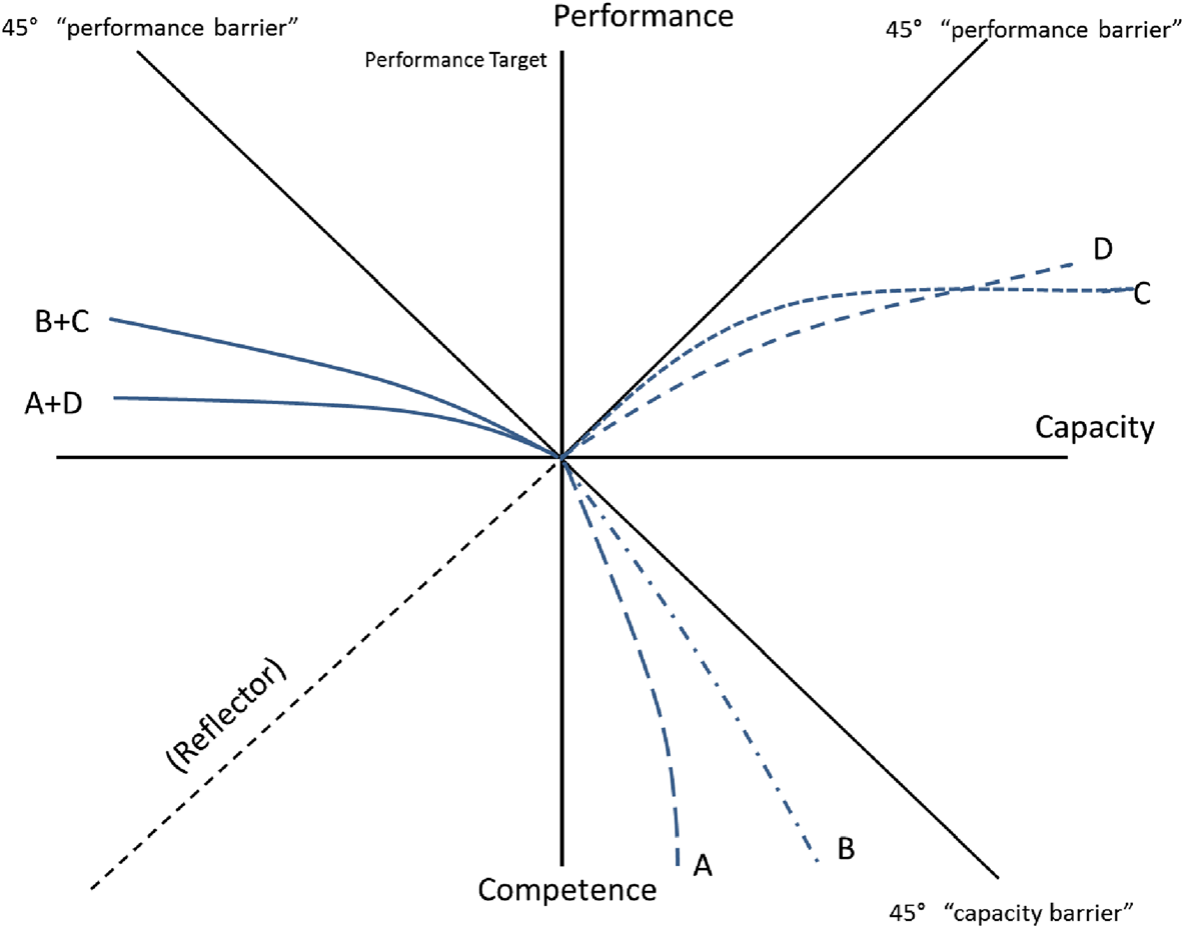
**Examples of possible relationships among competence, capacity, and performance.**

There is, to date, no evidence on the gaps and patterns in gaps at hospitals or in Liberia. Figure 4 shows a number of possible patterns using the three-gap framework. Pattern A demonstrates a significant gap between competence and capacity and is different from pattern B because after a certain point, increases in competence have no impact on capacity. Pattern B, in contrast, shows that when competence increases, capacity also increases. Pattern C demonstrates that for low levels of capacity, improvements in capacity lead to significant improvements in performance, but after a certain point, there is no further increase. Pattern D shows that performance increases with capacity at all levels, but not at a one-to-one rate.

The policy implications of these patterns are strikingly different. For example, pattern A suggests that training is of little use and that programs should focus on improving capacity. And pattern D suggests that improvement in capacity will lead to improvements in performance even if there is no improvement in motivation. These are only examples of possible patterns and the patterns in the lower right quadrant are independent of those in the upper right quadrant. Examining the upper left quadrant (the know-do gap), we would look at combinations of these patterns, and Figure 4 shows two possible combinations (A + D and B + C). Note that the implications of these patterns are different. If the true pattern is A + D, then training to improve competence is essentially pointless and it is far better to focus on improvements in infrastructure, equipment, and medicine to improve capacity and to focus as well on the motivation to improve effort.

#### Intensive facility evaluation

In order to measure the competence, capacity, and performance of health workers (doctors, clinicians, and nurses) at treatment and control facilities for selected hospitals, we will undergo an intensive facility evaluation at each facility at the baseline, midpoint (2.5 years) and final point (5 years). The evaluation is designed to measure the following:

1. The presence of health workers and their basic qualifications
2. Capacity and competence of health workers, using case studies and vignettes
3. Performance of health workers, using direct observation
4. Availability of key equipment and materials, using direct assessment by the research team
5. Structural assessment of facilities, using direct assessment by the research team
6. Patient satisfaction, using patient exit surveys
7. Health worker motivation, using health worker surveys.

It is not possible to measure the competence, capacity, or performance in all possible situations, so the evaluation will focus on the instruments as outlined in Table 2. The instruments used to measure these elements are available at https://sites.google.com/site/hfqualityassessment, on the page titled Hospital Quality Assessment.^a^Health worker motivation surveys are a blend of methodologies from [13,14].

**Table 2:** **Summary of instruments by service and location**

**Service**	**Location**	**Intensive evaluation**		
		**Direct observation**	**Competence/capacity**	**Exit interview**
Obstetrics				
Routine delivery	Labor ward	Labor and delivery	Partograph case study A	Maternal and neonatal care exit interview
			Partograph case study B	
Complicated delivery (PPH, sepsis, eclampsia)	Delivery room		PPH simulation	
Routine newborn care			Preeclampsia simulation	
Newborn asphyxia			Newborn health vignette	
			Newborn asphyxia vignette	
			Newborn resuscitation	
Post-delivery monitoring and discharge	Recovery ward	Labor and delivery recovery		
Pediatrics				
Routine pediatric care	Outpatient clinic	Pediatric fever, cough, diarrhea	Pediatric diarrhea vignette	Pediatric exit interview
			Pediatric fever vignette	
			Pediatric cough vignette	
Referred and emergency pediatric	Emergency intake	Pediatric fever, cough, diarrhea	Pediatric diarrhea vignette	
			Pediatric fever vignette	
			Pediatric cough vignette	
	Pediatric or general ward	Pediatric inpatient monitoring		
Emergency, inpatient, surgery				
Adult triage and emergency consultation	Emergency intake (nurse or MD)		Hernia A	General emergency and triage exit interview
			Hernia B	
			Small-bowel obstruction A	
			Small-bowel obstruction B	
			First-degree burn	
			Cholecystitis	
			Appendicitis	
	Consultation (MD)		Hernia A	
			Hernia B	
			Small-bowel obstruction A	
			Small-bowel obstruction B	
			First-degree burn	
			Third-degree burn	
			Cholecystitis	
			Appendicitis	
	Hospital ward	Post-surgical care and general ward		
Surgery	Surgical prep ward	Surgery		
	Operating theater			
	Recovery ward	Post-surgical care		
	OR cleanup	Post-surgical cleanup		
Facility				
	Staff	Facility staff roster	Staff motivation survey	
	Infrastructure	Facility equipment survey		

The actual measurement of competence, capacity, and performance comes from a series of yes or no questions on each instrument. For each condition, there is something that the health worker is supposed to do (take the patients temperature or check that the patient has signed a consent form, for example) and the member of the research team administering the case study, vignette, or direct observation vignette will indicate whether that thing was done. The competence, capacity, and performance score for each health worker is the percentage of items required or suggested by protocol that are actually administered. This follows the standard process for scoring direct observation and vignettes as outlined in [15,16].

Competence and capacity are measured for identical procedures and differentiated by the use of specific questions about the availability of equipment. For example, when assessing newborn care with the Newborn Health Simulation vignette, the health worker is asked “Please tell me, when a healthy baby is delivered, what care is important to give them immediately after birth and the first few hours thereafter?” One of the items that indicate competence is that they administer vitamin K. If the health worker indicates this procedure, the enumerator is instructed to verify that vitamin K is immediately available to the health worker. Saying they would administer vitamin K indicates competence, saying they would administer vitamin K together with having vitamin K present indicates capacity. Of course, the final test comes during direct observation when we indicate whether or not the health worker actually did administer vitamin K. In other cases, at the end of the case study, we ask “what equipment or materials would you have used if it had been available to you?”

Thus, we have designed specific elements of our survey to measure competence, capacity, and performance. These measures can be used for item-wise comparisons and overall health worker or facilities scores.

##### Item comparisons

In some cases, we can measure all three elements of the same task: does the health worker know that they are supposed to use vitamin K (competence), is vitamin K present (capacity), and is vitamin K used for newborns (performance)? These scores are dichotomous (yes/no) variables for all such items. These are the most direct test of the link between the three measures.

##### Overall scores for health workers and hospitals

In other cases (for example, the treatment of third-degree burns), we will not be able to measure performance. However, measures of competence or capacity for such items are still useful for measuring the competence of health workers or facilities, even if these scores cannot be directly compared to an average performance score for the same worker. The average competence, capacity, and performance scores are an average of the dichotomous variables over all applicable questions: for example, what proportion of the 45 measures of competence can a health worker appropriately describe? Because not every health worker will be examined for every available measure, these scores need to be adjusted for the difficulty of the items for which they were evaluated and we do this by subtracting the mean score for each item before summing scores across items. Thus,

$$ {Q}_i={\displaystyle \sum_{k=1}^N}\left({q}_{ik}-{\overline{q}}_k\right) $$

where *Q*_*i*_ is the score for each health worker or hospital, *q*_*i*k_ is a dichotomous variable indicated whether health worker *i* demonstrated competence, capacity or performance for item *k*, and ${\overline{q}}_k$ is the average score for item *k* across the whole sample. Thus, although we can report that a particular health worker performed 80% of the items that were required by protocol, a more useful number is that they performed 15 percentage points more than the average for all health workers who were evaluated for that item, for example.

Using the scores for competence, capacity, and performance, we can validate the selection of control facilities to match the treated facilities both by examining the levels of these three measures and by examining the relationships between these measures across types of facilities. In other words, is the pattern relating competence to capacity or relating capacity to performance similar across health workers in treated and control facilities?

### Augmented PBF

The HSSP focuses on rewarding the performance of hospitals as a whole, even though this performance is composed, for the most part, of the aggregated performance of individuals. The intensive evaluation at three points in time will help us understand how individual health workers are responding to the program and, to some degree, will help us separate the influence of (1) training, (2) infrastructure, equipment, and medicines, and (3) motivation. However, we cannot definitely understand the role of financial incentives in changing financial motivation as separate from improvement management and information flow.

In order to directly test the role of information within the context of a PBF program, we are developing a within-hospital information system that can be used to feed information to individual health workers about their effort and performance. Each quarter during the 5-year life of the program, a few health workers will be randomly selected into one of two information treatments. The treatments are designed as follows:

- *Control:* Information is collected from health registers on the quantity of services provided and key measures of performance
- *Own performance treatment:* For one quarter, the treated health worker is provided with a weekly summary of their own quantity and performance statistics.
- *Compared performance treatment:* For one quarter, the treated health worker is provided with a weekly summary of their own quantity and performance statistics compared to the average levels for all other health workers providing similar services.

All health workers will be part of the control until they are randomly selected to belong to one of the two treatments. At the conclusion of the quarter as a “treated” health worker, they will be given a choice to continue in their current treatment, switch to the other treatment, or return to the control^b^. We will, of course, test for long-term effects of treatment (beyond the quarter in which treatment was assigned).

The impact of treatment can be compared to performance before (for the same health worker) as well as to all other health workers who are not being treated. Thus, for each of the variables tested (see below), we will use a difference in difference strategy, comparing the change for a health worker who will be treated to their performance before treatment as well as to the performance of all other health workers in the control group.

The key element of the design is that we can control for changes that would have happened normally over time: both steady improvements and seasonal changes. Our treatment lasts only one quarter, but we will have data on many time periods before this quarter and after this quarter. This increases our statistical power and also allows us to understand the dynamics of the control group well as the post-treatment group [17,18]. Thus, we can include a specification in which we control for quarterly effects non-parametrically by including a dummy variable and coefficient for each quarter for all health workers in the facility. Thus, the outcome for health worker *i* in facility *j* at time *t* is a function of that health worker’s average outcome (*α*_*I*_), the flexible time effect for all health workers in facility *j*$\left(\overset{\rightharpoonup }{\beta_j}\ \overset{\rightharpoonup }{t}\right)$, and the treatment status of health worker *i* at time *t* (*ρT*_*it*_).

$$ {Y}_{ijt}={\alpha}_i+\overset{\rightharpoonup }{\beta_j}\ \overset{\rightharpoonup }{t}+\rho {T}_{it}+{\epsilon}_{it} $$

It is also possible that health workers have individual trends (improving or worsening over time) even after controlling for what is happening in the facility as a whole. In this case, we can only control for a linear time trend (*γ*_*i*_*t*), but this variable could potentially be important in understanding how health workers respond to the combination of PBF incentives and individual information.

$$ {Y}_{ijt}={\alpha}_i+\overset{\rightharpoonup }{\beta_j}\ \overset{\rightharpoonup }{t}+{\gamma}_it+\rho {T}_{it}+{\epsilon}_{it} $$

The exact outputs to be measured remains to be established, but at this stage, we propose the following measures (see [19] for examples of quality analysis using in-house register data):

- Quantity of services provided
  - Deliveries (vaginal and C-section), surgeries (by type)
- In-hospital outcomes
  - Post-surgical and post-delivery fevers in recovery ward
  - Mortality
- Re-admission rates following specific surgical procedures
- Evidence of medical errors and complications
  - Routine episiotomy
  - Perineal tears
  - Delay in initiating breastfeeding
  - Needle pricks
- Length of stay for well-understood illnesses
- Referral feedback completeness
- Data completeness
  - Surgical consent forms
  - Anesthesiology reports
  - Partograph forms

Hospitals in Liberia use a data collection system that tracks individual patients through the services provided during the course of their visit to a hospital, as well as across visits to the same hospital. The records of a patient’s visit are kept both in registers devoted to a room or service (e.g., intake, laboratory, and wound dressing), and on their medical record, stored in the hospital’s records room. Every patient has a unique patient number assigned when they first visit a facility and this number is used in all future visits.

The goal of the data collection exercise is to digitize select aspects of the paper data such that (1) multiple visits by the same patient can be linked and (2) statistics can be compiled about individual health workers who participate in the patient care. At each of the project hospitals, we will contract with a ministry of health employee to serve as the data entry clerk for the hospitals. These individuals have already been trained as data custodians but have not been asked to digitize any of the data. The advantage of using individuals who have already working in the system is that 1) they are familiar with the paperwork system and 2) they are already certified by the MoHSW to handle private patient information.

### Did the HSSP improve outcomes?

The ultimate goal of the HSSP is to increase the health of the population that uses the targeted health facilities. Such an outcome is difficult to measure directly but can be measured through the proxy outcome measures outlined in Table 1 above: (a) utilization, (b) satisfaction and trust, and (c) health outcomes, both for patients who use the hospital and for households in the communities that surround the hospital. These outcome measures are provided to estimate and describe the impact of the program. However, they are collected only at those facilities that are part of the project and therefore there are no controls.

There are three main sources of data on outcomes.

- *Hospital registers:* Data from hospital registers can be used to compare the numbers for different types of services over time. For example, by following individual cases over time, we can measure important outcomes, indicative of medical errors. In addition, retrospective case review can be used to measure the rate of medical errors, even when these errors do not lead to adverse outcomes. These outcomes are drawn from the same data set used for the augmented PBF.
- *Patient exit surveys:* While this data source is particularly useful for outpatient services, patients who received inpatient services can also be interviewed at patient discharge. Patients will be interviewed regarding the location they traveled from when they choose the facility, their satisfaction with services provided, and their trust in the services provided. This data is drawn from the exit interviews in the intensive facility evaluation.
- *Qualitative community utilization and opinion assessments:* In two communities within each hospital catchment area, we will conduct focus group interviews on attitudes towards the facility. We will deliberately create a focus group of women focusing on issues around antenatal care, childbirth, and postnatal care. As part of these focus groups, we will conduct short interviews with participants on recent utilization of the specific hospital.

## Trial status

The instruments have been piloted, and the baseline data for the treated facilities has been collected but has not been cleaned or analyzed. As the project was starting to collect baseline data in the control facilities, the MoHSW asked the evaluation team to pause our project so that they could reassign resources to the Ebola crisis and avoid unnecessary exposure. We expect to resume activities as the crisis ends and will have to re-evaluate the validity of the baseline data. It may be necessary to recollect this data to establish a new baseline and use the original baseline as a way of understanding the impact of the crisis on these hospitals.

## Endnotes

^a^The instruments benefitted from the contribution and previous works of teams at the World Bank working on programs in various countries, in particular the HRITF impact evaluation for the Kyrgyz Republic.

^b^Health workers who return to the control will not be included in the control comparison group because there may be long-term effects that alter their behavior even after the treatment has concluded.

## Abbreviations

GMRP: Graduate Medical Residency Program
GOL: Government of Liberia
HSSP: Health Systems Strengthening Project
MDG: Millennium Development Goal
MoHSW: Ministry of Health and Social Welfare (Liberia)
PBF: Performance-based financing
